# Targeting of Mcl-1 Expression by MiRNA-3614-5p Promotes Cell Apoptosis of Human Prostate Cancer Cells

**DOI:** 10.3390/ijms23084194

**Published:** 2022-04-11

**Authors:** Yi-Hsien Hsieh, Fang-Jung Yu, Yasser Nassef, Chung-Jung Liu, Yong-Syuan Chen, Ching-Yi Lin, Jia-Liang Feng, Min-Hua Wu

**Affiliations:** 1 Institute of Medicine, Chung Shan Medical University, Taichung 40201, Taiwan; hyhsien@csmu.edu.tw (Y.-H.H.); yasser@nassef.org (Y.N.); kevin810647@gmail.com (Y.-S.C.); 2 Department of Medical Research, Chung Shan Medical University Hospital, Taichung 40201, Taiwan; 3 Division of Gastroenterology, Department of Internal Medicine, Kaohsiung Medical University Hospital, Kaohsiung Medical University, Kaohsiung 80708, Taiwan; yufj@kmu.edu.tw (F.-J.Y.); 1020590@ms.kmug.org.tw (C.-J.L.); 4 Department of Medicine, Faculty of Medicine, College of Medicine, Kaohsiung Medical University, Kaohsiung 80708, Taiwan; 5 Regenetative Medicine and Cell Therapy Research Center, Kaohsiung Medical University, Kaohsiung 80708, Taiwan; 6 Division of Chest Medicine, Department of Internal Medicine, Taichung Veterans General Hospital, Taichung 40705, Taiwan; chingyii@vghtc.gov.tw; 7 Laboratory Department, Chung-Kang Branch, Cheng-Ching General Hospital, Taichung 40764, Taiwan; 8 Department of Medicinal Botanicals and Health Applications, Da-Yeh University, Chunghua 515006, Taiwan

**Keywords:** prostate cancer, growth, apoptosis, miRNA-3614-5p, Mcl-1

## Abstract

MicroRNA (miRNA) acts as a critical regulator of growth in various human malignancies. However, the role of miRNA-3614 in the progression of human prostate cancer remains unknown. In this study, our results demonstrated that miRNA-3614-5p exerts a significant inhibitory effect on cell viability and colony formation and induces sub-G1 cell cycle arrest and apoptosis in human prostate cancer cells. Myeloid cell leukemia-1 (Mcl-1) acts as a master regulator of cell survival. Using the miRNA databases, miRNA-3614-5p was found to regulate Mcl-1 expression by targeting positions of the Mcl-1-3′ UTR. The reduction of Mcl-1 expression by miRNA-3614-5p was further confirmed using an immunoblotting assay. Pro-apoptotic caspase-3 and poly (ADP-ribose) polymerase (PARP) were significantly activated by miRNA-3614-5p to generate cleaved caspase-3 (active caspase-3) and cleaved PARP (active PARP), accompanied by the inhibited Mcl-1 expression. These findings were the first to demonstrate the anti-growth effects of miRNA-3614-5p through downregulating Mcl-1 expression in human prostate cancer cells.

## 1. Introduction

Cancers of the urinary system accounted for 13.1% of the 19.3 million new cancers worldwide in 2020 [1]. Risk factors for the development of prostate cancer include longer life expectancy, diets high in red meat, inherited prostate-cancer susceptibility genes, and chronic inflammation [2]. The prognosis of prostate cancer depends on tumor stage, grade, pre-treatment prostate-specific antigen level, and Gleason score, which suggests that patients with localized or regional prostate cancer have a much higher survival rate than those with distant metastases [3]. Typically, the well-known modalities for cancer treatment include radiation therapy, surgery, hormone therapy, and cryosurgery [4]; however, these approaches are frequently associated with various adverse side effects [5]. Prostate cancer occurs due to the uncontrolled division of prostate cells, which in turn leads to abnormal cell growth and spread to other parts of the body [2]. Therefore, the identification of biomarkers and mechanisms involved in the uncontrolled growth of prostate cancer can help create novel strategies for treating this type of cancer.

Myeloid cell leukemia 1 (Mcl-1) is a member of the anti-apoptotic Bcl-2 family. Escape from cell death is one of the most prominent features of tumor cells and is closely associated with the dysregulation of members of the Bcl-2 family. Numerous knockout studies have demonstrated the importance of Mcl-1 and its anti-apoptotic function for normal cell development. Inducible deletion of Mcl-1 increases apoptosis and prevents development at the pro-B-cell and double-negative T-cell stages during early lymphocyte differentiation [6]. Conditional gene knockout demonstrates the important role of Mcl-1 in regulating the development of hematopoietic stem cells, hepatocytes, neutrophils, cardiomyocytes, and neurons [7]. The above-mentioned reports indicate Mcl-1 has a critical role in cellular health and development besides its established pro-survival function. However, human cancer cells containing amplifications of the Mcl-1 anti-apoptotic gene upregulate the Mcl-1 protein level for its survival and growth [8]. Suppression of Mcl-1 expression by norcantharidin is shown to induce mitochondrial-dependent apoptosis in human prostate cancer cells [9], which suggests that Mcl-1 plays an important role in regulating the progression of human prostate cancer. Suppression of Mcl-1 by norcantharidin induces mitochondrial-dependent apoptosis in human prostate cancer cells [9]. These findings suggested that Mcl-1 plays an important role in human prostate cancer progression.

MicroRNA (miRNA) acts as the critical regulator of growth in various human malignancies. miRNA biogenesis occurs in two locations. Intergenic miRNAs originate from non-coding regions between genes and are transcribed from unknown promoters [10]. Most miRNAs are considered to be intergenic miRNAs. A few miRNAs are termed intronic or intragenic miRNAs, which derive from non-coding introns or the 5′-UTR and 3′-UTR of host genes [11]. miRNAs are a class of small non-coding RNA molecules, about 21–25 nucleotides in length. miRNAs regulate the expression of their target genes through modifying post-transcription. miRNAs target the mRNA of its target genes in a sequence-specific manner through binding the 3′ untranslated regions (UTRs) of mRNAs and then result in mRNA degradation or translational repression [12]. More than 60% of all mammalian mRNAs are the targets of miRNAs [13]. Therefore, miRNAs regulate various cellular processes such as survival, growth and differentiation [14]. Specifically, miRNAs can act as tumor suppressors or oncogenes, which are involved in the development of human cancer [15]. miRNA-3614 is reported to be a tumor suppressor. The generation of miRNA-3614-3p and miRNA-3614-5p depends on the start from the 3′- or 5′-terminal of pre-miRNA. Inhibition of intragenic miRNA-3614-3p maturation by IGF2BP3 increases TRIM25 expression and promotes breast cancer cell proliferation [16]. miRNA-3614-5p is shown to inhibit the malignant behaviors of hepatocellular carcinoma (HCC) cells [17]. miRNA-3614-5p is a potential and novel prognostic biomarker for colorectal cancer [18]. Overexpression of miRNA-3614-5p attenuates cell proliferation and invasion of non-small-cell lung cancer (NSCLC) through down-regulating phosphoglycerate mutase 1 (PGAM1) expression [19]. In this study, we found that miRNA-3614-5p is less expressed in prostate cancer cells. miRNA-3614-5p is found to target Mcl-1 via analyzing the miRNA databases. miRNA-3614-5p overexpression inhibits Mcl-1 expression and increases Mcl-1-related pro-apoptotic caspase-3 and PARP proteins.

## 2. Results

### 2.1. Inhibitory Effect of MiRNA-3614-5p on Cell Viability in Human Prostate Cancer Cells

**Figure 1:**
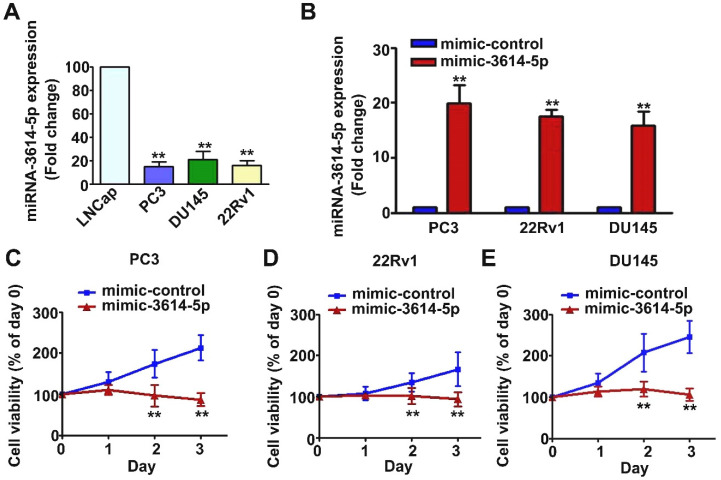
Effect of miRNA-3614-5p on the viability of human prostate cancer cells. (**A**) Endogenous expression of miRNA-3614-5p in human prostate cancer cells including LNCap, PC3, DU145 and 22Rv1 cell lines. (**B**) Exogenous expression of miRNA-3614-5p in human PC3, DU145 and 22Rv1 prostate cancer cell lines transfected with mimic-3614-5p. Cells transfected with mimic-control as the control group. (**C**–**E**) Cell viability of human PC3, DU145 and 22Rv1 prostate cancer cell lines transfected with mimic-control or mimic-3614-5p for days 1, 2 and 3 were measured using an MTT assay. **, *p* < 0.01 versus control (mean ± SE, *n* = 3). mimic-3614-5p as mimic miRNA-3614-5p.

Endogenous levels of miRNA-3614-5p in human LNCap, PC3, DU145 and 22Rv1 prostate cancer cell lines were measured. PC3, DU145 and 22Rv1 cells presented with lower levels of expression of miRNA-3614-5p, as shown in Figure 1A. To identify the role of miRNA-3614-5p in the progression of human prostate cancer, human PC3, DU145 and 22Rv1 cell lines were transfected with mimic-3614-5p to overexpress exogenous miRNA-3614-5p, as shown in Figure 1B. The influence of miRNA-3614-5p on cell viability was analyzed in human PC3, DU145 and 22Rv1 prostate cancer cells. After 6 h of transfection with mimic-3614-5p, these prostate cancer cells were cultured for another 24 h, 48 h and 72 h, and then cellular viability was analyzed using an MTT assay. Cell viability was significantly reduced by mimic-3614-5p in PC3, DU145 and 22Rv1 cell lines at 48 h and 72 h compared with mimic-control (Figure 1C–E).

### 2.2. Inhibitory Effect of MiRNA-3614-5p on Colony Formation in Human Prostate Cancer Cells

**Figure 2:**
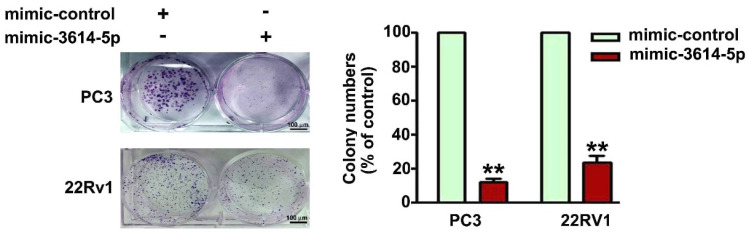
Effect of miRNA-3614-5p on colony formation of human PC3 and 22Rv1 prostate cancer cells. The cell growth rate of human PC3 and 22Rv1 prostate cancer cells transfected with mimic-control or mimic-3614-5p for 1 week was measured using a colony formation assay. Cells transfected with mimic-control as the control group. **, *p* < 0.01 versus control (mean ± SE, *n* = 3). Mimic-3614-5p as mimic miRNA-3614-5p. Scale bar, 100 μm.

The cell growth rate was further measured using a colony formation assay to confirm the inhibitory effect of miRNA-3614-5p on the proliferation of PC3 and 22Rv1 cells transfected with mimic-control or mimic-3614-5p for 1 week. Cells transfected with mimic-control were considered the control group (Figure 2). These findings revealed that miRNA-3614-5p exerted a suppressive effect on the growth of human prostate cancer cells.

### 2.3. Induction of Sub-G1 Cell Cycle Arrest by MiRNA-3614-5p in Human PC3 and 22Rv1 Prostate Cancer Cells

**Figure 3:**
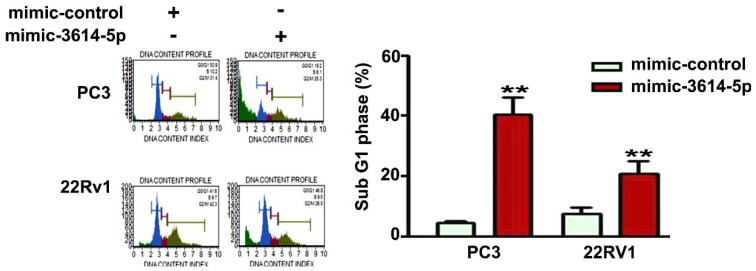
Effect of miRNA-3614-5p on cell cycle arrest in human PC3 and 22Rv1 prostate cancer cells. Regulation of cell cycle distribution in human PC3 and 22Rv1 prostate cancer cells transfected with mimic-control or mimic-3614-5p were analyzed by using PI staining through flow cytometry. Cells transfected with mimic-control as the control group. **, *p* < 0.01 versus control (mean ± SE, *n* = 3). DNA content index (x-axis): analyzes the cell populations in each phase of the cycle (sub G1, G0/G1, S, G2/M phase); DNA content (y-axis): measures the percentage of cells in each cell cycle phase. Sub-G1: apoptotic cells. Mimic-3614-5p as mimic miRNA-3614-5p.

Human PC3 and 22Rv1 prostate cancer cells were transfected with mimic-control or mimic-3614-5p for 48 h. Cell cycle distribution of PC3 and 22Rv1 cells further were analyzed with a PI staining assay by using a flow cytometry system. The results revealed that miRNA-3614-5p induced a significantly high percentage of the cell cycle to stop at the sub-G1 phase (Figure 3).

### 2.4. Induction of Apoptosis by MiRNA-3614-5p in Human PC3 and 22Rv1 Prostate Cancer Cells

**Figure 4:**
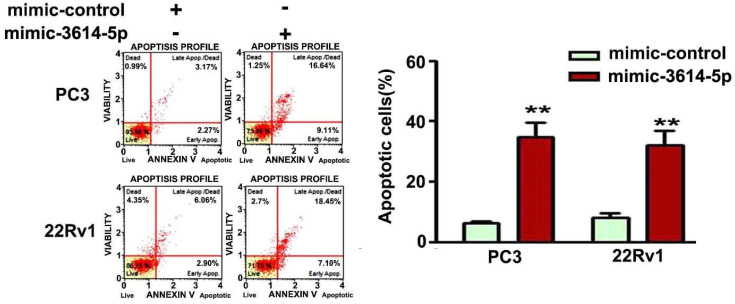
Effect of miRNA-3614-5p on apoptosis induction in human PC3 and 22Rv1 prostate cancer cells. Regulation of apoptosis induction in human PC3 and 22Rv1 prostate cancer cells transfected with mimic-control or mimic-3614-5p were analyzed by Annexin V/PI staining through flow cytometry. Mimic-control as the control group. **, *p* < 0.01 versus control (mean ± SE, *n* = 3). Y-axis presents the cell viability (%) of normal healthy cells (LL), early and apoptotic cells (LR/UR) and necrotic cells (UL). Mimic-3614-5p as mimic miRNA-3614-5p.

The induction of cell apoptosis by miRNA-3614-5p was further analyzed in PC3 and 22Rv1 cells with Annexin/PI staining through a flow cytometry system. After 48 h of transfection with mimic-control or mimic-3614-5p, the apoptotic cells were significantly induced due to high miRNA-3614-5p expression in human PC3 and 22Rv1 prostate cancer cells. These results demonstrated that miRNA-3614-5p may induce apoptotic cell death in human prostate cancer cells (Figure 4).

### 2.5. MiRNA-3614-5p Is a Crucial Targeting on Mcl-1 in Human PC3 and 22Rv1 Prostate Cancer Cells

**Figure 5:**
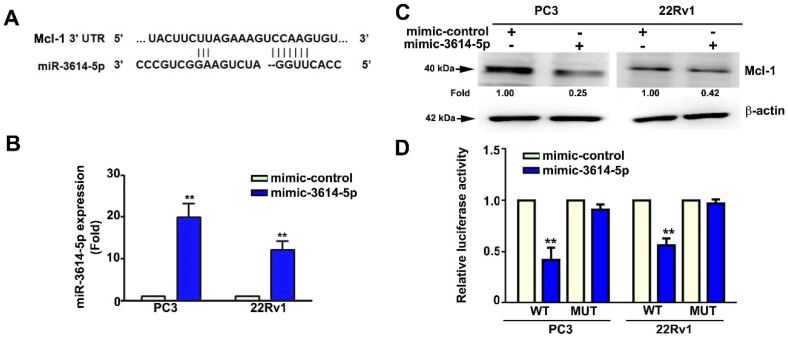
Effect of miRNA-3614-5p on Mcl-1 expression in human PC3 and 22Rv1 prostate cancer cells. (**A**) The miRNA-3614-5p targeting sites on the 3′ UTR of Mcl-1. (**B**) The expression of miRNA-3614-5p in human PC3 and 22Rv1 prostate cancer cells transfected with mimic-control or mimic-3614-5p were measured by qRT-PCR assay, with GAPDH as a loading control. (**C**) The level of Mcl-1 protein in human PC3 and 22Rv1 prostate cancer cells transfected with mimic-control or mimic-3614-5p were measured by immunoblotting assay. β-actin was used as a protein loading control. (**D**) Luciferase activity of the luciferase reporter constructs containing either the WT or Mut-3′-UTR of Mcl-1 promoter following mimic-control or mimic-3614-5p transfection in human PC3 and 22Rv1 prostate cancer cells. **, *p* < 0.01 versus control (mean ± SE, *n* = 3). Mimic-3614-5p as mimic miRNA-3614-5p.

To clarify the role of Mcl-1 in prostate cancer cells, we utilized the knockdown assay for Mcl-1 in human PC3 and 22Rv1 cells. We found that knockdown Mcl-1 inhibited endogenous Mcl-1 protein expression (Supplementary Figure S1A) and significantly decreased the viability of PC3 and 22Rv1 cells (Supplementary Figure S1B). Using the miRNA databases, miRNA-3614-5p was found to regulate Mcl-1 expression by targeting positions of the Mcl-1-3′ UTR (Figure 5A). To examine the transfection efficiency of miRNA-3614-5p in both PC3 and 22Rv1 cells by qRT-PCR assay, a high level of miRNA-3614-5p in mimic-3614-5p-treated cells was found compared with mimic-control treated cells (Figure 5B). Regulation of Mcl-1 expression by miRNA-3614-5p was further investigated by immunoblotting analysis in human PC3 and 22Rv1 prostate cancer cells. The results showed that human PC3 and 22Rv1 cells transfected with mimic-3614-5p had significantly lower levels of Mcl-1 than those transfected with mimic-control. Cells transfected with mimic-control were considered the control group. (Figure 5C). To understand whether miRNA-3614-5p directly targets the 3′-UTR of Mcl-1 promoter, we constructed luciferase reporter plasmids containing either wild type (WT) or mutated (Mut) versions of the 3′-UTR of the Mcl-1 promoter. Our results found that the mimic-3614-5p inhibited the luciferase activity of Mcl-1 (WT)-3′-UTR promoter by luciferase reporter assays; however, it did not reduce the luciferase activity in Mcl-1 (Mut)-3′-UTR promoter (Figure 5D). These results demonstrated an important miRNA-3614-5p binding site on the Mcl-1 3′-UTR promoter that induces cell apoptosis.

### 2.6. Induction of Pro-Apoptotic Caspase-3 and PARP Expressions by MiRNA-3614-5p in Human PC3 and 22Rv1 Prostate Cancer Cells

**Figure 6:**
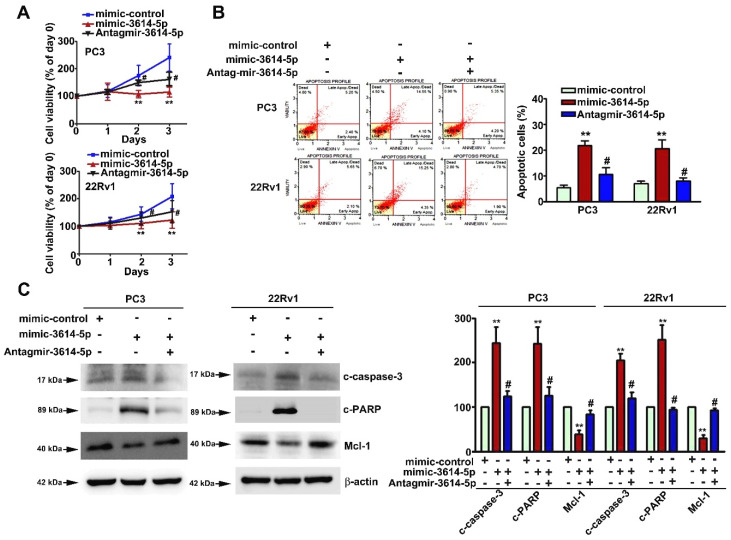
Effect of miRNA-3614-5p on cell viability, cell death and the expression of apoptotic-related proteins of human prostate cancer cells. Human PC3 and 22Rv1 cells were transfected with mimic-control or mimic-3614-5p for 48 h. (**A**) Cell viability was detected with MTT assay. (**B**) Apoptotic cells were measured with Annexin V/PI staining by flow cytometry. (**C**) The expression of cleaved caspase-3 (c-caspase-3), cleaved PARP (*c-PARP*) and Mcl-1 proteins were measured by immunoblotting analysis. Cells transfected with *mimic-control* as the control group. β-actin as a protein loading control. **, *p* < 0.01 versus control (mean ± SE, *n* = 3), #, *p* < 0.05 versus mimic-3614-5p.

Mcl-1 was reported to act as a critical regulator of apoptosis in various human malignancies. We further explored the mechanism behind miRNA-3614-5p-reduced Mcl-1 in human prostate cancer cells. Human PC3 and 22Rv1 prostate cancer cells were transfected with mimic-control or mimic-3614-5p for 48 h and then were harvested to measure the expression of Mcl-1-regulated pro-apoptotic proteins in prostate cancer cells. Active forms of caspase 3 (c-caspase-3) and PARP (c-PARP) were then measured by conducting an immunoblotting assay. We found that mimic-3614-5p significantly increased the expression of c-caspase-3 and c-PARP, accompanied by a decrease in Mcl-1 (Figure 6). These results demonstrated that miRNA-3614-5p induces cell death in human prostate cancer cells.

## 3. Discussion

**Figure 7:**
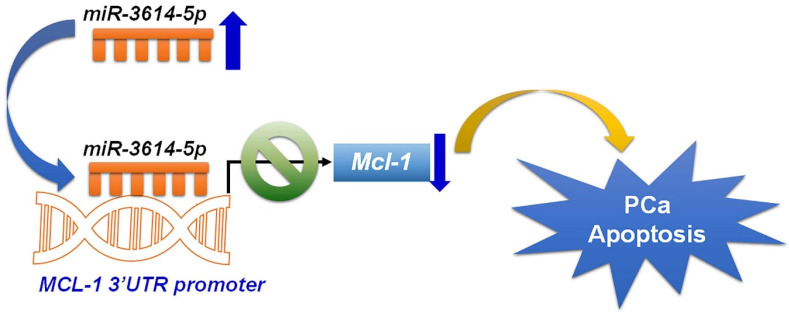
Schematic illustration of the overexpression of miRNA-3614-5p targeting Mcl-1 3′-UTR and inhibition of Mcl-1 expression, leading to an apoptotic effect on human prostate cancer cells.

Prostate cancer is the most commonly diagnosed urologic cancer and is one of the leading causes of cancer deaths in men. The exploration of anti-cancer mechanisms, the identification of novel molecule biomarkers, and the application of potential treatment methods are critical issues against prostate cancer. In this study, we found that the expression of miRNA-3614-5p is lower in human prostate PC3, DU145 and 22Rv1 cancer cells. Results from cell viability, colony formation, cell cycle distribution and apoptosis detection assays confirmed the critical role of miRNA-3614-5p in regulating the growth of human prostate cancer cells. miRNA-3614-5p may be considered a biomarker for early diagnosis and detection of prostate cancer. The anti-cancer mechanism of miRNA-3614-5p is further disclosed by using the miRNA databases. Anti-apoptotic Mcl-1 was found to be targeted by miRNA-3614-5p, which reveals that prostate cancer cells with lower miRNA-3614-5p expression have higher malignant properties. Our findings highlight the crucial role of miRNA-3614-5p in regulating Mcl-1 anti-apoptotic signaling in human prostate cancer cells (Figure 7). Thus, the antitumor properties of miRNA-3614-5p may be used as a therapeutic molecule in prostate cancer.

miRNAs closely regulate cellular progressions in human prostate cancer. Norcantharidin induces mitochondrial-dependent apoptosis via epigenetic upregulation of miRNA-320d in human prostate cancer cells [9]. Loss of miRNA-135b favors the incidence of bone metastases by significantly increasing the migratory capacity of prostate cancer cells [20]. miRNA-629-5p plays an oncogenic role by promoting prostate cancer development and metastasis by targeting A-kinase anchor protein 13 (AKAP13) [21]. miRNA-195-5p regulates cell proliferation, migration and invasion via regulating mind bomb 1 (MIB1) in prostate cancer cells [22]. miRNA-21 overexpression increases cell proliferation and migration, as well as the levels of insulin receptor substrate 1 (IRS1), sterol regulatory element-binding protein 1 (SREBP-1), fatty acid synthase (FASN) and acetyl-CoA carboxylase (ACC) in human prostate cancer cells [23]. miRNA-877-5p is decreased in prostate cancer tissues and cells. Overexpression of miR-877-5p exerts tumor suppressor properties by inhibiting the malignant progression of prostate cancer by directly targeting sperm-specific antigen 2 (SSFA2) [24]. miRNA-642a-5p acts as a tumor suppressor by targeting Wilms Tumor 1 (WT1) gene and cell-cycle progression in prostate cancer [25]. miRNA-145-5p upregulates apoptosis and inhibits the migration, invasion, and metastasis of prostate cancer via directing phospholipase D 5 (PLD5) modulation [26]. In this study, we found that miRNA-3614-5p is expressed at low levels in human prostate cancer cells. miRNA-3614-5p overexpression suppresses cell growth, increases sub-G1 cell cycle arrest and induces apoptosis in PC3, DU145 and 22Rv1 cells.

The unique expression domains, targets, and gain-and-loss phenotypes of specific miRNAs have important implications for cell fate determination. miRNAs regulate cell fate by modifying the proliferation of cells by targeting oncogenes or cell cycle regulators in normal cells [14]. Intracellular miRNA expression patterns are reported to influence the necrotic and apoptotic cell death fates [27]. The most highly expressed miRNAs in normal prostate differ from those in prostate cancer tissues and prostate cancer cell lines [28]. Loss of control of miRNAs expression in normal cells may lead to the development of a tumor microenvironment and tumorigenesis. Studies reveal that the reduced expression of miR-205, miR-31, miR-145, and the miR-17-92 cluster are frequently observed in prostate cancer. The loss of basal cells (loss in miR-205, miR-31, and miR-17HG), reorganization of the stroma (reduction in miR-145) and overexpression of miR-375 in cancerous luminal cells contribute to prostate tumorigenesis [28]. Anti-apoptotic Mcl-1 is downregulated by miRNA-3614-5p tumor suppressor. Reducing Mcl-1 by miRNA-3614-5p overexpression may result in cellular apoptosis in normal cells. In this study, we observed that miRNA-3614-5p is less expressed in prostate cancer cells. Anti-apoptotic Mcl-1 downregulated by miRNA-3614-5p suggests that loss of miRNA-3614-5p may contribute to prostate tumorigenesis. miRNA-3614-5p may play a critical role in the transformation of normal cells into cancer cells.

Mcl-1 is reported to be highly amplified in human cancers [8]. The dramatic effect of perturbing Mcl-1 dependence on tumor development underscores the need for tumor cells to maintain Mcl-1 expression and stability. Mcl-1 knockdown induces apoptosis through upregulating caspase-3 and -7 and PARP and releasing Smac/DIABLO and Omi/HtrA2 into the cytoplasm. Furthermore, Mcl-1 knockdown also induces cell cycle arrest via decreasing cyclin D1, cell division cycle gene 2 (cdc2), and cyclin-dependent kinases 4/6 in gastric cancer [29]. Knockdown of Mcl-1 expression by miRNA-101 inhibits cell survival and proliferation and increases the sensitivity of human A549 lung cancer cells to etoposide [30]. miRNA-26b suppresses tumorigenicity and promotes apoptosis by targeting the Mcl-1 protein in small-cell lung cancer (SCLC) cells [31]. In this study, we found that Mcl-1 is targeted by miRNA-3614-5p. Mcl-1 downregulation by miRNA-3614-5p induces the activation of caspase 3 and PARP in prostate cancer cells. Nowadays, miRNA is considered a novel therapeutic approach to target prostate cancer progression [32]. Two main strategies, including intra-tumoral (local) and systemic delivery, are considered for the application of miRNA mimics or anti-miRs. Systemic delivery of synthetic miRNA-16 inhibits the growth of metastatic prostate tumors via downregulation of multiple cell-cycle genes [33]. The chitosan nanoparticle-mediated delivery of miRNA-34a inducts autophagy and apoptosis by downregulation of MET and Axl and c-Myc, which then lead to the inhibition of tumor growth and metastasis preservation of bone integrity in vivo [34]. The cationic polymer nanoparticle-mediated delivery of miR-124 inhibits the proliferation, motility, and colony formation of prostate cancer cells [35]. Upregulation of miR-155-5p by selenium nanoparticles inhibits tumor metastasis of prostate cancer through targeting IκB kinase ε and Sma- and Mad-related protein 2 [36]. miR-217 and miR-181b-5p enhance chemosensitivity and apoptosis in advanced prostate cancer [37]. In this study, we found that delivery of miRNA-3614-5p inhibits growth and induces sub-G1 cell cycle arrest and apoptosis in prostate cancer cells. Suppression of Mcl-1 expression by miRNA-3614-5p significantly leads to apoptosis through activating caspase-3 and PARP. These results suggest that miRNA-3614-5p acts as a novel therapeutic target for the inhibition of prostate cancer progression.

## 4. Materials and Methods

### 4.1. Chemical and Reagent

3-(4,5-Dimethylthiazol-2-yl)-2,5-diphenyl-tetrazolium bromide (MTT) was obtained from Sigma-Aldrich (St. Louis, MO, USA). Primary antibodies against cleaved caspase-3, and cleaved PARP were purchased from Cell Signaling Technology Inc. (Danvers, MA, USA). Mcl-1, β-actin, HRP-mouse and HRP-rabbit antibodies were purchased from Santa Cruz Biotechnology (Santa Cruz, CA, USA).

### 4.2. Cell Lines

Human LNCap, PC3, DU145 and 22Rv1 prostate cancer cell lines were purchased from Bioresources Collection and Research Center, Food Industry Research and Development Institute (Hsinchu, Taiwan). LNCap, PC3 and 22Rv1 cells were maintained in RPMI 1640 medium, and DU145 cells were maintained in MEM medium with 10% FBS containing 100 U/mL penicillin, 10 mM HEPES, 0.1 mM NEAA and 1 mM sodium pyruvate. The cultures were incubated at 37 °C in a humidified atmosphere of 5% CO_2_. Cells were passaged every 2~3 days to obtain exponential growth.

### 4.3. Cell Proliferation Assay

To determine the effect of miRNA-3614-5p on cell viability, human PC3, DU145 and 22Rv1 prostate cancer cells were seeded with a density of 4 × 10^4^ cells per well in 24-well plates (Greiner Bio-one, Frickenhausen, Germany) and transfected with miRNA-3614-5p for 24, 48 or 72 h. The medium was replaced with fresh cell culture medium containing 0.5 mg/mL MTT (Sigma-Aldrich, St. Louis, MO, USA) for 4 h. The formazan crystals were dissolved in DMSO (100 μL/well). Cell viability was measured at 570 nm using a Multiskan MS ELISA reader (Labsystems, Helsinki, Finland) as previously described [38].

### 4.4. Cell Cycle Distribution by Flow Cytometry

To measure the cell cycle, human PC3 and 22Rv1 prostate cancer cells (2 × 10^5^ cells/well) transfected with miRNA-3614-5p for 48 h were fixed with 75% ice ethanol overnight. Cells were then washed in PBS and stained with propidium iodide (PI) reagent for 20 min. DNA content and outcome data were measured through flow cytometry using the Muse Cell Analyzer (Merck Millipore, Burlington, MA, USA) as previously described [39].

### 4.5. Colony Formation Assay

Human PC3 and 22Rv1 prostate cancer cells were seeded into 6-well plates (5 × 10^3^/well) and transfected with miRNA-3614-5p for 2 weeks. More than 100 colonies were stained with 0.5% crystal violet solution for 30 min at room temperature and counted as previously described [40].

### 4.6. Annexin V/PI Staining by Flow Cytometry Analysis

To perform an apoptosis assay detecting early and late apoptotic statuses, human PC3 and 22Rv1 prostate cancer cells (2 × 10^5^ cells/well) transfected with miRNA-3614-5p for 48 h were harvested and analyzed by using the Muse Annexin V and Dead Cell Assay Kit (Merck Millipore). Briefly, cells were incubated with 5 μL of Annexin V-FITC and 5 μL of PI reagent at room temperature in darkness for 15 min. The apoptotic cell population was then analyzed by the Muse Cell Analyzer (Merck Millipore) as described in a previous study [40].

### 4.7. Western Blotting Analysis

Equal amounts of total protein (20 μg) from human PC3 and 22Rv1 prostate cancer cells were subjected to 10~12% SDS-PAGE for protein separation and then transferred onto PVDF membrane (Life Technologies, Carlsbad, CA, USA). The membranes were blocked with 5% nonfat dry milk in Tris-buffered saline with Tween 20. The blocked membranes were further incubated with the primary target antibodies and subsequently with secondary antibodies to detect antibody-bound protein bands by using the Luminescent Image Analyzer (LAS 4000 mini, GE Healthcare Bio-Sciences, Pittsburgh, PA, USA) as described in a previous study [9].

### 4.8. MiRNA Prediction

To identify the target gene of miRNA-3614-5p, the miRbase [41] and TargetScan [42] programs were used to predict putative miRNAs binding sites in the 3′UTR of human Mcl-1 (NM_021960).

### 4.9. MiRNA Transient Transfection

Human prostate cancer cells cultured on 6-cm dishes were then incubated with RNAiMAX Transfection Reagent (Thermo Fisher Scientific, Waltham, MA, USA) with mimic-3614-5p (5′-CCACUUGGAUCUGAAGGCUGCCC-3′) or mimic-control (5′-UCACAACCUCCUAGAAAGAGUAGA-3′) for 6 h, respectively, and added to the fresh culture medium and incubated for 48 h. Then, these cells were harvested and analyzed by an immunoblotting assay. These mimic miRNA and negative controls were all synthesized and designed by GenDiscovey Biotechnology Inc. (New Taipei City, Taiwan).

### 4.10. Dual-Luciferase Reporter Assay

PC3 and 22Rv1 cells were seeded in 6-cm culture dishes overnight, then cotransfected with pGL4.13-Mcl-1-3′UTR-WT/Mutant promoter and miRNA-3614-5p by using RNAiMAX Transfection Reagent (Thermo Fisher Scientific, Waltham, MA, USA) for 48 h. The 3′-UTR promoter sequences of Mcl-1 containing the miRNA-3614-5p binding site were detected by qPCR assay. The Mcl-1-3′UTR mutant promoter sequences were as follows: forward, 5′-CAATTCCTACAGCTTTCCCCTGCCAT-3; reverse, 5′-ATGGCAGGGGAAAGCTGTAGGAATTG-3′. The Dual-Luciferase Reporter Assay System (Promega E1910) was then used to detect the luciferase activity of Mcl-1-3′UTR promoter following the manufacturer’s instructions.

### 4.11. Statistical Analysis

Data were compared between the two groups using an unpaired two-tailed Student’s t-test. All data are expressed as the mean ± standard deviation of triplicate experiments. Statistical significance was defined as *p* < 0.01 as described.

## Data Availability

The authors will freely release all data underlying the published paper upon direct request to the corresponding author.

## Supplementary Materials

The following supporting information can be downloaded at: https://www.mdpi.com/article/10.3390/ijms23084194/s1.
