# Supplementary material for: Targeting of Mcl-1 Expression by MiRNA-3614-5p Promotes Cell Apoptosis of Human Prostate Cancer Cells
Source: Int J Mol Sci. 2022 Apr 11;23(8):4194. doi: 10.3390/ijms23084194 (PMC9029607; doi:10.3390/ijms23084194)
Supplement: Supplementary file 1 [file ijms-23-04194-s001.zip › ijms-1622727-supplementary.pdf]

# Targeting of Mcl-1 expression by miR-3614-5p promotes cell apoptosis of human prostate cancer cells

**Yi-Hsien Hsieh, Fang-Jung Yu, Yasser Nassef, Chung-Jung Liu, Yong-Syuan Chen, Ching-Yi Lin, Jia-Liang Feng, Min-Hua Wu**

## Supplementary Figure S1

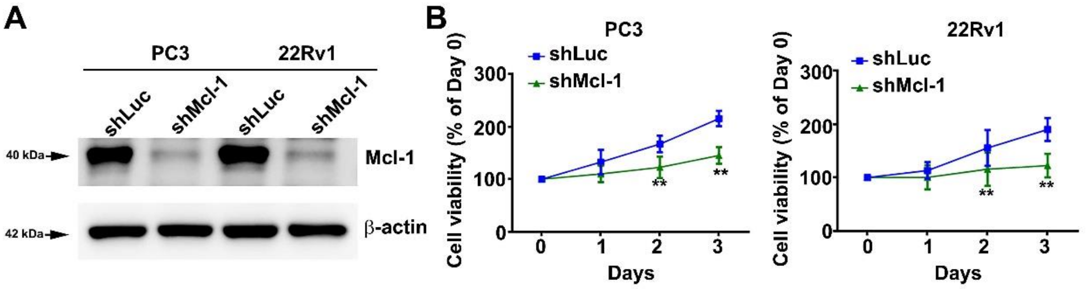


**Supplementary Figure S1**: Knockdown of Mcl-1 on cell viability in human PC3 and 22Rv1 prostate cancer cells. (A) The Mcl-1 protein expression of knockdown Mcl-1 in human PC3 and 22Rv1 prostate cancer cells by immunoblotting assay. (B) Cell viability were measured with MTT assay. β-actin as a protein loading control. **, $p < 0.01$ versus shLuc cells or Day 0 (cell viability), (Mean ± SE, n = 3).

.
